# Multispectral Image Feature Points

**DOI:** 10.3390/s120912661

**Published:** 2012-09-17

**Authors:** Cristhian Aguilera, Fernando Barrera, Felipe Lumbreras, Angel D. Sappa, Ricardo Toledo

**Affiliations:** 1 Department of Electrical and Electronics Engineering, Collao 1202, University of Bío-Bío, 4051381 Concepción, Chile; 2 Computer Vision Center, Edifici O, Campus UAB, 08193 Bellaterra, Barcelona, Spain; E-Mails: jfbarrera@cvc.uab.es (F.B.); felipe@cvc.uab.es (F.L.); angel.sappa@cvc.uab.es (A.D.S.); ricardo@cvc.uab.es (R.T.); 3 Computer Science Department, Edifici O, Campus UAB, 08193 Bellaterra, Barcelona, Spain

**Keywords:** multispectral image descriptor, color and infrared images, feature point descriptor

## Abstract

Far-Infrared and Visible Spectrum images. It allows matching interest points on images of the same scene but acquired in different spectral bands. Initially, points of interest are detected on both images through a SIFT-like based scale space representation. Then, these points are characterized using an Edge Oriented Histogram (EOH) descriptor. Finally, points of interest from multispectral images are matched by finding nearest couples using the information from the descriptor. The provided experimental results and comparisons with similar methods show both the validity of the proposed approach as well as the improvements it offers with respect to the current state-of-the-art.

## 1. Introduction

The analysis of multispectral or multiband imaging has recently attracted the attention of the research community for applications in the areas of image and video processing (e.g., [1–4]). The information provided by the images from different spectral bands, either directly or supplementary to the information provided by the visible spectrum, can help tackle different problems in an efficient way. However, the processing of images from spectral bands outside the visible spectrum requires the development of new tools, or the adaptation of current ones, opening new challenges for the image and video processing community. Multispectral analysis has been widely studied in the remote sensing field, where satellite images are commonly registered and fused. Recently, due to advances in the technology, multispectral imaging is being used in applications such as video surveillance or driver assistance (e.g., [5–7]); just to mention a few; where visible (VS: 0.4–0.7 μm) images are merged with Near-Infrared (NIR: 0.75–1.4 μm), Short-Wave Infrared (SWIR: 1.4–3 μm), Mid-Wave Infrared (MWIR: 3–8 μm) or Long-Wave Infrared (LWIR: 8–15 μm) ones.

Feature points are at the base of different computer vision problems and are generally studied at three levels—feature point: detection, descriptor and matching. One of the most successful and widely used approaches is the SIFT algorithm [8]. Over the last decade several variations of the original algorithm, as well as some novel approaches, have been proposed focusing on the perceived weaknesses of SIFT (e.g., [9,10]). All the approaches mentioned before are intended for applications that involve images from the same spectral band, generally Visible Spectrum (VS) images. Recently, applications that combine feature points from different spectral band images are being developed. These works are mainly based on the use of classical SIFT algorithm, or minor modifications to the classical approach. For instance [11] proposes a scale restriction criteria in order to reduce the number of incorrect matches of SIFT when it is adopted to tackle the multispectral case. Accurate matching results have been reported when the spectral bands of the pair of images are somehow near (VS-NIR); however, further improvements are needed for tackling those cases where the spectral bands are far away from each other (VS-LWIR). Actually, recent studies have shown that as the spectral bands go away from visible spectrum, classical feature descriptors generally used for finding matching and registration of images in the visible spectrum (e.g., SIFT, SURF, *etc.*) are useless (e.g., [12,13]).

**Figure 1.**
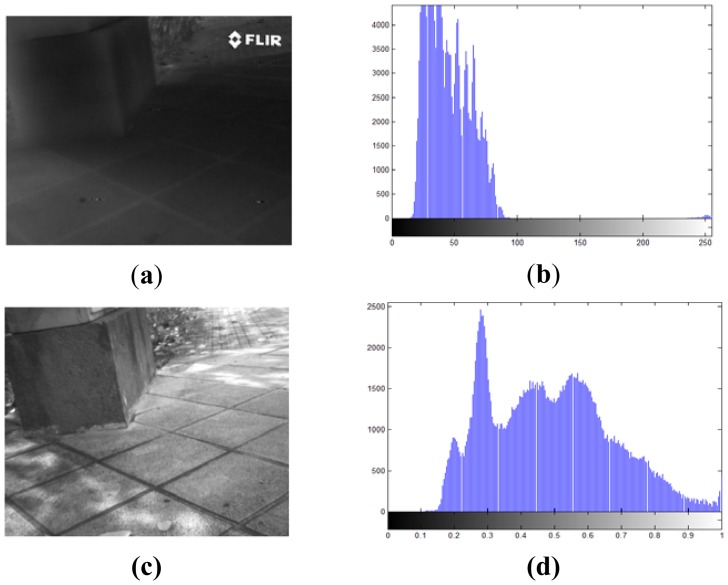
(**a**) LWIR image; (**b**) Histogram of LWIR image; (**c**) VS image; (**d**) Histogram of VS image.

The difficulty in finding correspondences between feature points from VS-LWIR images results from the nonlinear relationship between pixel intensities. Variations in LWIR intensities are related to variations in the temperature of the objects, while variations in VS intensities come from color object and light reflections. Therefore, this nonlinear relationship results in a lack of correlation between their respective gradients. Furthermore, LWIR images appear smoother, with loss of detail and texture [14], so that the detection of corners, as candidates for local descriptor points, is also poorly favored. In conclusion, most of the image processing tools that use gradient of pixels based descriptors need to be adapted, or otherwise they become useless. Figure 1 shows a couple of images, from the same scenario, obtained with a camera working in the visible spectrum and a camera in the infrared spectrum; their corresponding histograms are provided in the right column showing the lack of contrast in the infrared image as well as showing how some details are missed. In addition to the lack of contrast and missed details it can be observed that in the infrared image there is a big difference in the transformation of intensities with respect to the one presented in the visible spectrum. This fact becomes critical to define the method that best describes this kind of images, since the transformations of intensity pixels in the infrared spectrum are non linear or non correlated with respect to the corresponding visible ones. Additionally, it should be noticed that in the LWIR images other kinds of information is available, which is not present in the visible one. The latter is an important characteristic where there is not enough illumination in the given scene, for instance when driving a car at night (e.g., [6,15]).

From the three levels mentioned above—feature point: detection, descriptor and matching—the feature point descriptor becomes *the key element* when images from VS and LWIR spectrum are considered. Even though the percentage of correct matching can be improved by introducing modifications at the matching stage, results remain very poor when SIFT, or modifications of it (e.g., [11,16]), are used as descriptors in the multispectral case (VS-LWIR), as will be presented in the Experimental Results section. This low correspondence rate is mainly due to the lack of descriptive capability of gradient in LWIR images, which in general appears smoother with loss of detail and texture. Actually, even in the cases where the detected feature points correspond to the same position in both images, the matching results remain quite poor due to the differences in their gradient orientation, which is used as a descriptor by SIFT. In other words, since the descriptors are by nature different it is not correct to try to use them for finding similarities and matches.

## 2. Proposed Approach

**Figure 2.**
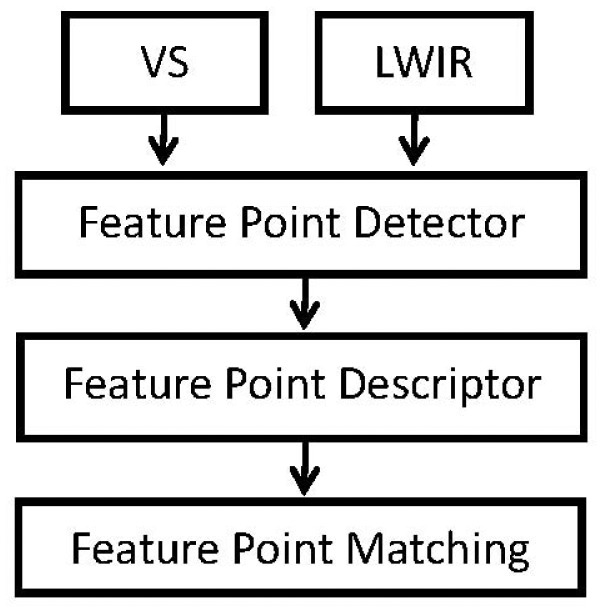
Flowchart of the proposed approach.

The proposed scheme consists of a scale-space pyramid, like the one used by SIFT. Similarly, invariant features are used, but by modifying the feature vector in such a way to incorporate spatial information from the contours of each keypoint without using gradient information. This allows us to generate a correlated parameter space in both the VS and LWIR images. Our proposal uses a descriptor based on the edge histogram. This edge orientation histogram describes the shapes and contours from LWIR images, keeping in the scale-space their invariance. Figure 2 presents a flow chart of the proposed method. It consists of three steps, namely detection, description and matching, as detailed next.

### 2.1. Feature Point Detection

Feature points, which will be also referred to as keypoints, are detected by using a scale-space pyramidal representation. They correspond to the maxima and minima of a difference of Gaussiass applied over a series of smoothed and resampled images [8]. The result from this first stage is a set of stable keypoints, similar to those resulting from classical SIFT. Note that this result is invariant to scale, position and orientation, which are needed for registration applications. In the current implementation potential keypoints are obtained by setting the SIFT like detector with the following parameters: sigma = 1.2 and threshold = 40. The same parameters' setting is used in both multispectral images resulting in a set of **P**_VS_ and **P**_LWIR_ keypoints. Each keypoint is denoted by a vector (*x_i_, y_i_, σ_i_*), where (*x_i_, y_i_*) correspond to the location and (*σ_i_*) is the scale of that pyramidal representation where the keypoint appears.

### 2.2. Feature Point Description

**Figure 3.**
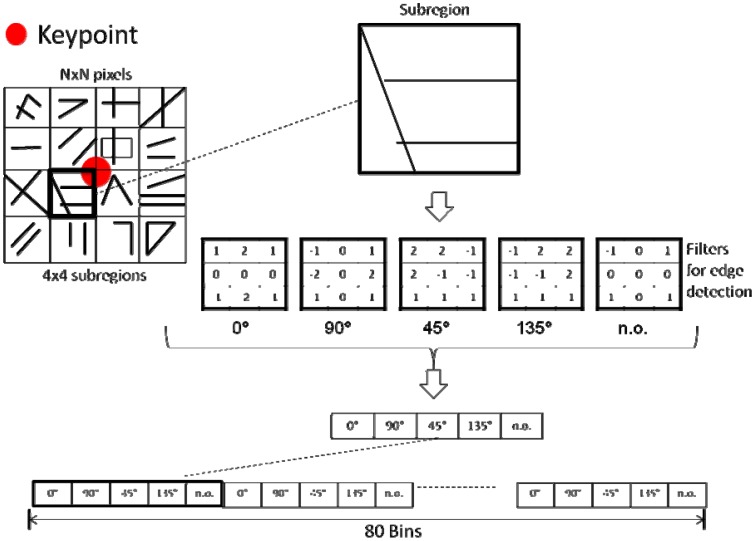
Proposed EOH based keypoint image descriptor for VS-LWIR images.

Detected feature points are described through the use of an Edge-Oriented-Histogram, which is the main contribution of current work and will be referred to henceforth as EOH. These EOHs incorporate spatial information from the contours in the neighbourhood of each feature point. They describe the shapes and contours from both VS and LWIR images. The idea behind the proposed descriptor is motivated by the nonlinear relationships between image intensities so that the descriptor should be mainly based on region information instead of pixel information. Hence, the proposed descriptor is based on the use of histograms of contours' orientations in the neighbourhood of the given keypoints. Initially, both images (VS and LWIR) are represented by means of their edges, which are extracted using the Canny edge detector algorithm [17]. In all the cases Canny's thresholds have been automatically set relative to the highest value of the gradient magnitude of the image and σ = 4. Once both images are represented by means of their edges, feature points detected in Section 2.1 are described as illustrated in Figure 3. The different steps of the feature point descriptor are detailed below.

Firstly, a region of N × N pixels, centered at the given keypoint, is obtained. Then, this region is split up into 4 × 4 = 16 subregions. Finally, each one of these subregions is represented by a histogram of contours computed following the Edge Histogram Descriptor (EHD) [18] of the MPEG-7 standard [19]. This histogram represents the spatial distribution of four directional edges and one non-directional edge (five bins in total); these bins correspond to contour orientations of 0, 45, 90, and 135 degrees; additionally a bin with no orientation (n.o.) is considered, which corresponds to those areas that do not contain a contour. Every pixel of each subregion contributes to a bin of the histogram according to the five filters, of 3 × 3 pixels, shown in Figure 3. The filter with the largest value is used as a criterion to vote in the corresponding bin. After processing all the elements of the 16 subregions a vector with 80 elements is obtained (16 × 5 = 80). This vector is normalized (by dividing each one of its components by the Euclidean norm) and used as the vector of characteristics, which will be also referred to as the descriptor vector for every keypoint.

The selection of the right window size (N) for every keypoint is an important factor of the proposed scheme. A window with a too small or big size will increase the number of wrong matching of keypoints. Table 1 presents the performance of the proposed approach when windows with different sizes are considered to compute the vector of characteristics mentioned above; these values represent the average obtained with the whole data set, which contains 100 pair of multispectral images. It can be seen that the best performance is obtained when windows with sizes in between 80 × 80 and 100 × 100 are used.

### 2.3. Feature Point Matching

This stage finds nearest keypoints from different spectral images, in the descriptor space by filtering feature vectors with low descriptive elements. This process is based on the Euclidean distance between the corresponding descriptor vectors. Like the SIFT algorithm, in order to increase the matching robustness, two keypoints are matched only if the ratio between the first and second best matches is smaller than a given threshold. Additionally, the matching robustness is increased by discarding those keypoints that have some of their subregions without information (*i.e.*, subregions only containing a few contours); finally, the scale restriction proposed in [11], and used in [16], is also considered in the current work to improve the performance of the proposed approach. This scale restriction process consists in discarding incorrect matches using the scale difference (*SD*) of the given pair of keypoints *P_VS_(x_VS_,y_VS_,σ_VS_)* and *P_LWIR_(x_LWIR_,y_LWIR_,σ_LWIR_)*:

(1) $$SD(P_{VS},P_{\text{LWIR}})=\sigma_{VS}-\sigma_{\text{LWIR}}$$

as proposed in [11], the match is rejected if it does not satisfy the following scale restriction criteria:

(2) a<SD<b

where the values *a* and *b* are obtained by first computing a histogram of *SDs* of all matches; then, the peak in that *SDs* histogram, which is noted as $\bar{SD}$, is extracted and used to define the sough values:

(3) $$\begin{array}{cc} a=\bar{SD}-0.9 & b=\bar{SD}+0.9 \end{array}$$

**Figure 4.**
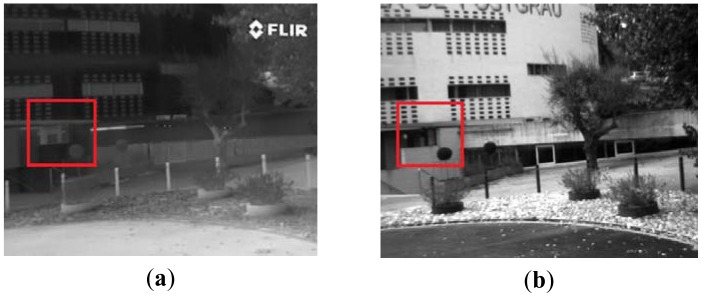

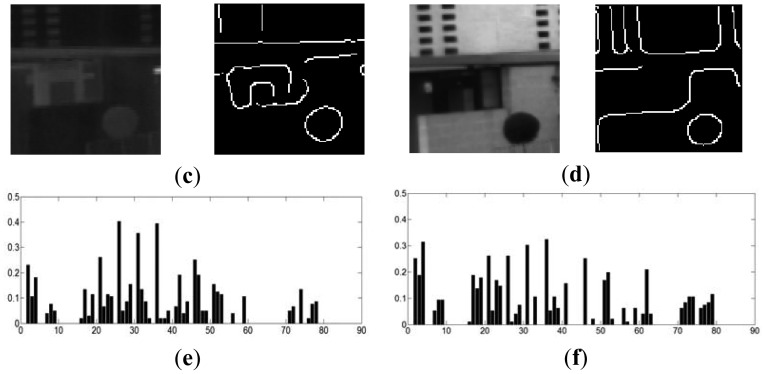
Steps of the proposed VS-LWIR keypoint description method: (**a**) and (**b**) keypoints on VS and LWIR images together with their corresponding neighborhoods; (**c**) and (**d**) images of contours of the given keypoints; (**e**) and (**f**) histograms used as descriptor vectors.

Figure 4 shows the different steps of the proposed algorithm for a pair of keypoints from VS and LWIR images.

## 3. Experimental Results

The proposed approach has been evaluated with a data set containing 100 pairs of VS-LWIR images. These images have been obtained using the cameras detailed in Table 2.

**Figure 5.**
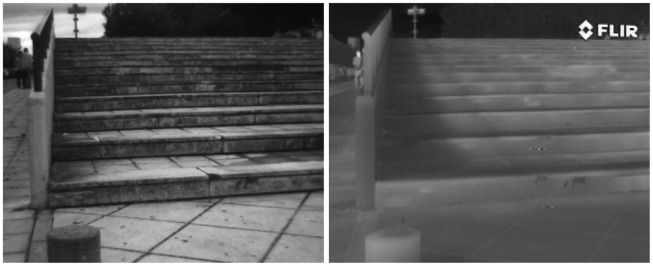

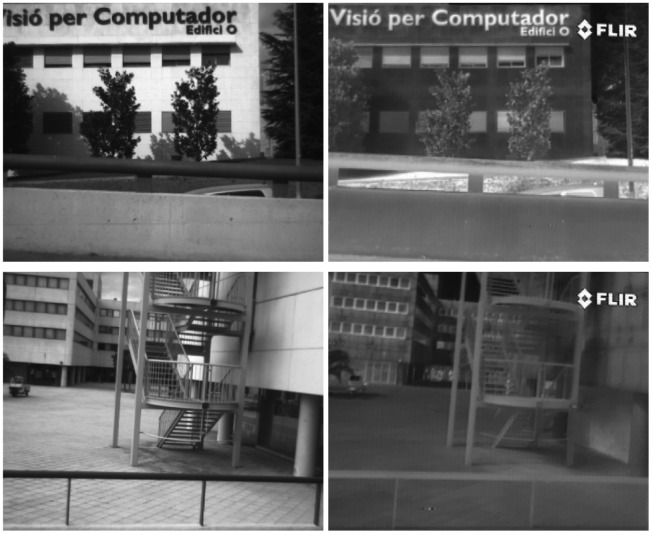
Illustrations of some of the VS-LWIR image pairs from the evaluated data set, which contains 100 pairs.

In order to make the comparisons easier, in all the cases the images were rectified and aligned so that matches should be found in horizontal lines. This rectification and alignment process is just applied to facilitate the evaluation of the performance of the proposed approach as well as for further comparisons with other algorithms. The data set contains outdoor images of different urban scenarios. Figure 5 shows three pairs of multispectral images contained in the data set.

This data set is available through our website (http://www.cvc.uab.es/adas/projects/simeve/) for evaluation and comparison with other multispectral feature point detectors and descriptors. The performance of the proposed approach is evaluated with a Precision and Recall scheme. Furthermore, results are compared with other implementations. Table 3 shows average results, computed over the whole data set, obtained with different descriptors; we can appreciate that the proposed approach has the best performance when compared with all the other methods.

**Figure 6.**
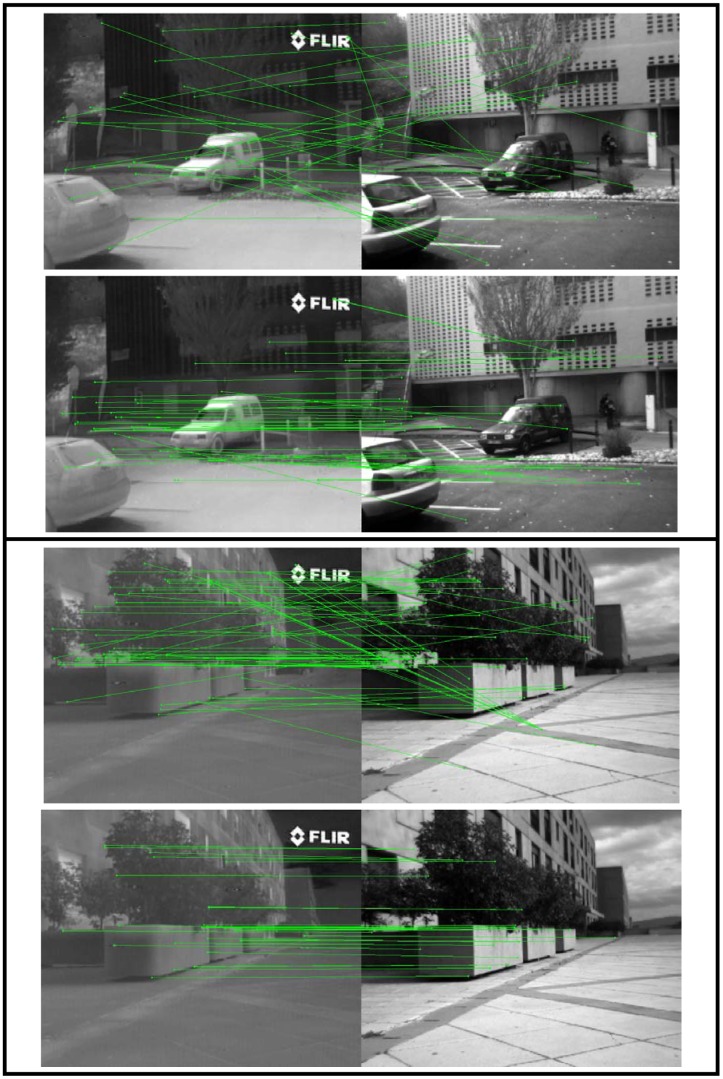
Keypoints matched (green segments) in two image pairs (the whole data set contains 100 pairs of **VS**-LWIR images) using: (**top**) SIFT descriptor; (**bottom**) proposed EOH based descriptor.

Figure 6 just illustrates the results obtained with only two pairs of VS-LWIR images using both SIFT and the proposed EOH based descriptors (quantitative evaluation over the whole data set is presented in Table 3). Note that since these pairs correspond to rectified images the matching should correspond to keypoints lying in the same row; in other words the segments that connect keypoints should be horizontal lines.

It can be appreciated that in the first case (top illustration) SIFT only matches correctly a few keypoints (two points), from a total of 34 keypoints, which represents about 5% success. On the contrary, when the proposed EOH based approach is considered, 36 keypoints, from a total of 51, are correctly matched (about 68% success). In the second case (bottom illustration), the classical SIFT algorithm matches 32 keypoints, from a total of 69 keypoints (46% success); while the proposed approach reaches the 90% success (28 keypoints are correctly matched from a total of 31).

**Figure 7.**
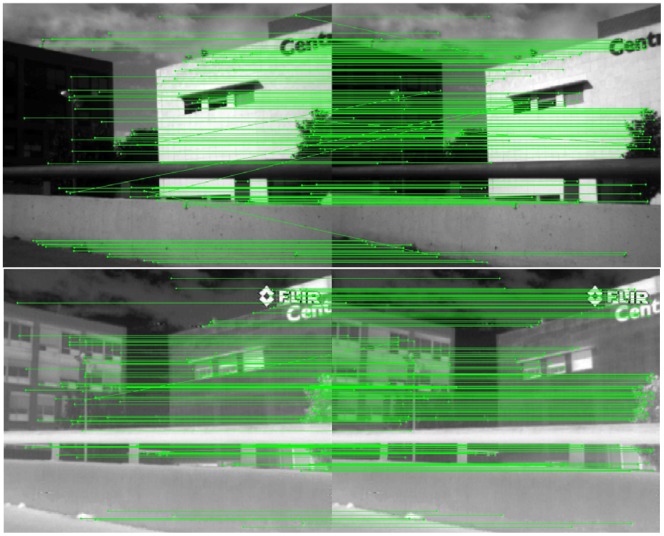
Illustrations of the results obtained with the proposed approach when images from the same spectral band are considered: (**top**) VS-VS; (**bottom**) LWIR-LWIR.

Finally, although out of the scope of current work, Figure 7 shows how the proposed approach can also be used as a feature point descriptor when images from the same spectral band are considered. In these illustrations in can be seen that the proposed approach reaches 86.2% of success when a pair of VS-VS images is considered and 84.6% of success in the LWIR-LWIR case. Note that these two cases are just presented as illustrations; more rigorous evaluations and comparisons are needed, over a large set of image pairs, to obtain more solid conclusions.

## 4. Conclusions

A novel multispectral feature descriptor method is presented, which is useful to register VS-LWIR images. It is based on the use of a SIFT-like detector to extract feature points. Then, an Edge Oriented Histogram (EOH) based approach is proposed as a robust descriptor for characterizing multispectral keypoints. Finally, matches are obtained by finding nearest couples in the feature description space. The proposed approach has been evaluated with a large set of multispectral pairs of images and compared with the current state-of-the-art. Finally, it is shown that the proposed approach could also be also used when pairs of images from the same spectral band are considered. Future work will focus on studying the use of more elaborated edge descriptor techniques in order to improve the matching percentages.

## Figures and Tables

**Table 1.** Average correct matching from the whole data set; images of 408 × 506 pixels.

**Window Size**	**20 × 20**	**40 × 40**	**60 × 60**	**80 × 80**	**100 × 100**	**120 × 120**	**140 × 140**
SIFT	6%	6%	6%	6%	6%	6%	6%
EOH-SIFT	2%	17%	27%	36%	37%	35%	32%

**Table 2.** Camera specifications.

**Specifications**	**VS**	**LWIR**

Image sensor type	CCD	Thermal
Resolution	640 × 480	320 × 240
Wavelength	0.4 to 0.7 μm	8 to 14 μm
Focal length	6 mm	19 mm

**Table 3.** Average (1-Precision) and Recall for 100 VS-LWIR images.

**VS\LWIR**	***Recall***	***(1-Precision)***

SIFT [8]	6%	93%
SURF [10]	45%	96%
GOM-SIFT [11]	14%	86%
OR-SIFT [16]	5%	97%
*Proposed Approach*	**74%**	**59%**
